# Interaction Network Prediction and Analysis of Anorexia Nervosa

**Published:** 2019

**Authors:** Majid REZAEI TAVIRANI, Mostafa REZAEI TAVIRANI, Reza VAFAEE

**Affiliations:** 1 Faculty of Medicine, Iran University of Medical Sciences, Tehran, Iran; 2 Proteomics Research Center, Shahid Beheshti University of Medical Sciences, Tehran, Iran

**Keywords:** Anorexia nervosa, Eating disorder, Protein-protein interaction network analysis, Protein clustering, Gene ontology

## Abstract

**Objectives:**

Anorexia Nervosa (AN) as a mental condition is a common eating disorder among young women. We aimed to shed lights on molecular behavior of this serious disorder in terms of protein interacting profile to provide further insight about its complexity.

**Materials & Methods:**

The AN related genes were extracted from STRING database and included in interactome via Cytoscape software. The central nodes of the network were enriched via gene ontology (GO) by ClueGO+CluePedia and the action relationship between the nodes were determined by CluePedia.

**Results:**

Six genes including *LEP, INS, POMC, GCG, SST, *and* ALB* were introduced as hub-bottlenecks that among them *LEP, INS, *and* POMC* were the super hub-bottlenecks based on further analysis. Action map analysis showed prominent role of hubs relative to bottlenecks in the network. Regulation of behavior, regulation of carbohydrate biosynthetic process, and regulation of appetite are the top associated processes for the identified hub genes.

**Conclusion:**

Topological analysis proposed the five hub-bottlenecks as the most central genes in the network, these genes and their contributing biological terms may suggest additional importance in AN pathogenesis and thereby possible candidates for therapeutic usage. However, further studies are required to justify these findings.

## Introduction

The eating disorder, anorexia nervosa (AN) accounts for the highest rate of death among the other psychiatric disorders (1). Its name became common from the late 19^th^ century. About 0.9% and 0.3% of women and men in the USA, respectively are diagnosed with AN. Patients with this disorder have an altered body shape since they fear to gain weight, and therefore not eating properly. About 90% of AN cases are women (2). In addition, the rate of this disorder is increasing in young women with unidentified etiology (3, 4). 

Moreover, the management of this heterogeneous eating disorder is allied with complications (5) as it shows resistance to the available treatments (6). In this regard, one of the important changes in AN patients is hormonal level alterations that can induce low bone mineral density (BMD) with irreversible consequences even after weight restoration (7). 

The interaction between genetic and environmental factors are responsible for this disorder (8). Most of the molecular studies on AN, are limited to genetic, genome-wide and transcriptome studies. The corresponding genetic and genome-wide studies are very heterogeneous and no candidate genes showed significantly replicated and related. Additionally, none of the genes was significant at expression levels (9). A recent proteomic study on a mouse model indicated that the inflammation in hypothalamus was associated with AN and obesity (10). 

Despite the presence of the conducted molecular studies, the underlying molecular mechanisms was remained elusive (11). One of the widespread used new disciplines in molecular studies is protein-protein interaction (PPI) network analysis used for highlighting genes with further vital roles in terms of centrality properties. The role of these contributors is in the network integrity and function that may be associated with the abnormal phenotype or a disease condition (12). In fact, any small changes in these critical elements may trigger vast amount of interaction changes in the whole interacting profile (13). 

Therefore, introducing a panel of such main genes may be beneficial for disease molecular basis understanding and consequently deciphering the underlying mechanisms (14). On the other hand, PPI network analysis of AN has been remained to study. In this regard, introduced AN genes by previous studies and available in String database were evaluated as a protein-protein interaction network for determination of the most central ones in the onset and development of anorexia disorder. 

## Material and Methods

In our study, Cytoscape 3.4.0 visualizes the protein-protein interaction network for anorexia. In a way that, STRING DB V 10.5 (http://string-db.org/), Plug-in was applied for interaction query for the studied disorder. This application provides three different sources for the interaction mapping including protein query, PubMed query, and disease query (15). By using of disease database here, the interaction profile of AN was retrieved. The all known genes related to AN from the sources linked to STRING were detected as query genes. The confidence score for interactions was set to 0.5 and other information for genes such as their relevant tissues was also provided. Furthermore, the centrality analysis was performed by the usage of Network Analyzer, well-integrated in Cytoscape (16). Two important parameters including degree and betweenness centrality are considered for the network examination by the mentioned software. The nodes with highest values of degree are known as hubs similarly nodes with largest amount of betweenness centrality are called bottlenecks. Those nodes with the top rank of these quantities are identified as hub-bottlenecks designated as the central genes in the PPI network. For hub-bottleneck element selection, the top 10% of hubs and bottlenecks were examined and the common nodes were selected.

Following the network construction, CluePedia assessed the action type between the identified hub genes. The action interpretation is extracted from STRING Action File available in CluePedia panel. The queried action types for our hubs were activation, expression, and inhibition coded with different colors. Action scoring was based on kappa statistics which is customizable from 0 to 1 and it can be shown as thick and thin lines. Here, it was set to 0.5 (medium) cut off for any kinds of actions. In addition, for assessing functional annotation of the hub genes, ClueGO+CluePedia was applied. In this algorithm, the linked biological processes (BPs) and molecular function (MF) for the hubs are presented as groups of terms. The terms are interconnected with the assigned kappa score. The statistical analysis used here was as follows:

 Kappa score = 0.5 Corrected *P*-value< 0.05 

Number of genes per term =3 Percentage for the queried terms =4

Grouping level: Min= 2, Max= 8 

Bonferroni step down was the used test for *P*-value correction. Moreover, two-sided (enrichment/depletion) tests based on hypergeometric distribution for terms and groups were selected (17, 18). 

## Results

For the network query, the number of requested nodes was set to 300 while only 250 nodes and 1170 edges were obtained. Among these 250 nodes, 75 were isolated. Therefore, the main connected component of this network consists of 148 nodes and 1147 edges. This component was considered for the centrality analysis. The important centrality values including degree and betweenness centrality were analyzed. The determined hubs and hub-bottlenecks are shown in Table 1.

**Table 1:** The list of 10% of highest degree nodes (hubs). The nodes with the asterisk sign indicate Hub-bottlenecks that are the top common genes between hubs and bottlenecks. Disease score; DS and Betweenness centrality; BC

**Row**	**Name**	**Description**	**DS**	**Degree**	**BC**
1	LEP*	Leptin	3	69	0.13
2	INS*	Insulin	2.02	68	0.12
3	POMC*	Proopiomelanocortin	2.22	65	0.07
4	*NPY*	Neuropeptide Y	2.34	58	0.03
5	GCG*	Glucagon	1	54	0.05
6	SST*	Somatostatin	1.41	51	0.04
7	*NPS*	Neuropeptide S	2.03	49	0.02
8	*CRH*	Corticotropin releasing hormone	2.25	48	0.04
9	ALB*	Albumin	1.35	45	0.10
10	*CCK*	Cholecystokinin	1.93	40	0.007
11	*TRH*	Thyrotropin-releasing hormone	1.60	40	0.009
12	*IL6*	Interleukin 6 (interferon, beta 2)	0.8	39	0.04
13	*IGF1*	Insulin-like growth factor 1 (somatomedin C)	2.60	38	0.02
14	*OXT*	Oxytocin/neurophysin I prepropeptide	1.92	38	0.01
15	*GAL*	Galanin/GMAP prepropeptide	1.34	38	0.01

**Figure 1:**
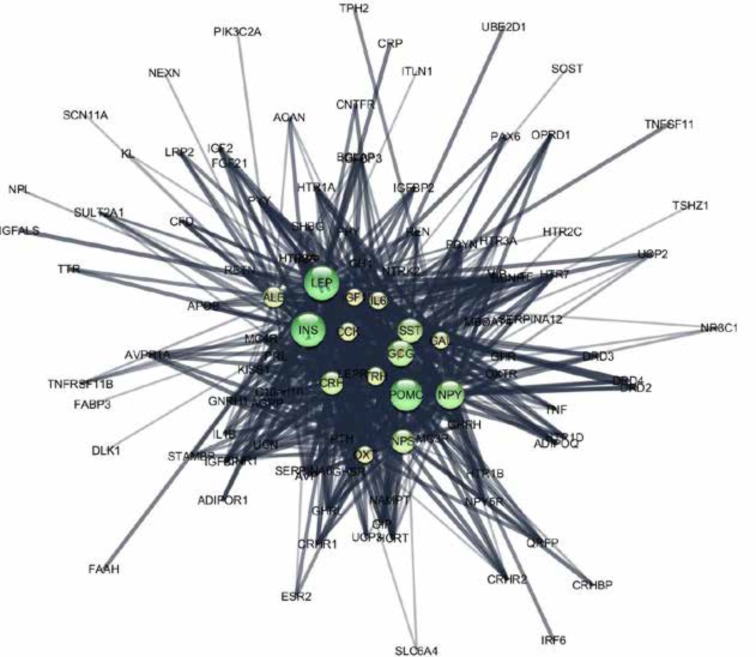
A backbone network analysis of the initial network shows that this network consists of 109 nodes and 642 edges. This evaluation express that this network is about 74% similar to the main network. The most central hub-bottleneck genes in the backbone network are introduced as super hub-bottlenecks

A backbone network derived from the identified hubs in Table 1, is created by selecting the hubs and their interconnections to determine to how much extent the initial constructed network was dependent on the contribution of these central elements. This network is constructed by selecting hubs and their interactions and consequently re-analyzing them based on centrality (Figure 1).

**Figure 2:**
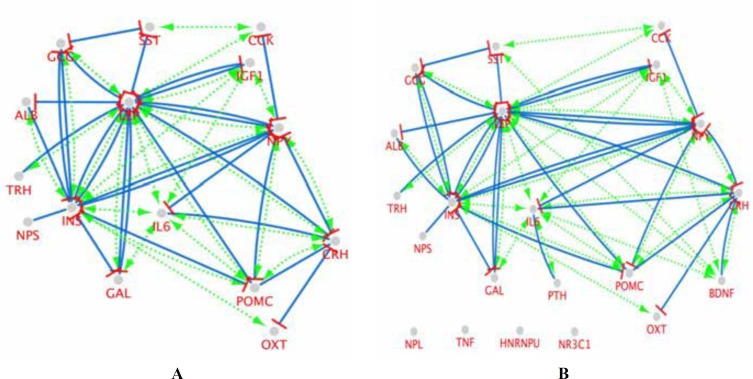
Nested pathway network view extracted via CluePedia, STRING Action File YU. The green, blue, and red colors indicate activation, expression, and inhibition, respectively. The cut off for these action scores was set to 0.5, **A:** a network of hub genes, all the genes are interacting with each other. **B:** A network of hubs and bottlenecks, four elements including *NPL, TNF, HNRNPU*, and *NR3C1* as bottlenecks are isolated in this map. Only two of six bottlenecks (*BDNF* and *PTH*) were included in the network

Interaction type analysis could provide further knowledge about the hubs and bottlenecks interaction type and simultaneously their pivotal role in the network. At first, a network of hub nodes (15 genes) (Figure 2-A) was constructed to analysis them in this matter and then another network including hubs and bottlenecks (21 genes) (Figure 2-B) was made as a validation test to better interpret the attribution of super hub-bottlenecks in the network foundation. 

The examined nested pathway networks showed more information about the behavior of hubs and bottlenecks. In this sense, the associated data for isolated bottleneck nodes in Figure 2-B are tabulated in Table 2. 

**Table 2:** A list of isolated bottlenecks in Figure 2-B and their relative betweenness centrality, degree, and ranks of BC and degree amounts

**Name**	**BC**	**Rank of BC**	**Degree**	**Rank of degree**
*NR3C1*	0.06	5	12	69
*NPL*	0.04	10	3	105
*TNF*	0.04	12	18	52
*HNRNPU*	0.03	14	4	103

**Figure 3:**
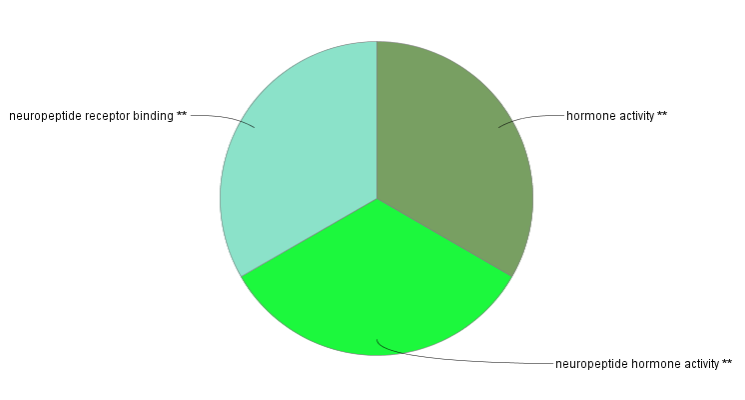
The Pie chart view of main molecular functions related to the hubs, the different groups are shown with individual colors. The two asterisk sign indicates to *P*-value <0.001

**Figure 4:**
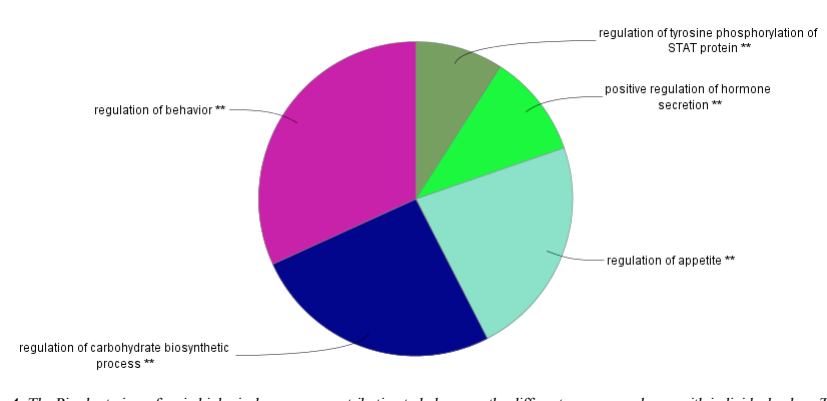
The Pie chart view of main biological processes contributing to hub genes, the different groups are shown with individual colors. The two asterisk sign indicates to *P*-value <0.001

The enrichment analysis for the hub nodes was based on molecular functions and biological processes that were performed by ClueGO from GO Database (Figures 3 and 4).

## Discussion

As anorexia is one of the complex and lethal mental conditions (19), studying molecular profile can help to better understand its underlying mechanisms (20, 21). One of the new concepts that provide more information with regard to interaction pattern of molecular basis is analyzing PPI map (22). Here, STRING DB, Cytoscape application, carried out the examination of anorexia interactome profile based on disease query. Since the data are extracted from the different data sets linked to STRING, the reported data are widespread and can be considered for human species. The obtained network showed involvement of many genes with the specific contributing disease scores. The participation of these elements was further studied in regard to the centrality properties in the constructed network.

By evaluating the hubs and bottleneck genes based on our designed method, six genes including *LEP, INS,* *POMC, GCG, SST,* and *ALB* were identified as hub-bottlenecks. Among these central nodes,* LEP, INS*, and *POMC* showed the highest values as it is further analyzed and visualized as a backbone network in Figure 1. This network implies on super-hub-bottleneck properties of these three genes. The initial network integrity is considerably depended on the presence of the hubs. Further investigation implied on the significant roles of hubs and with a special respect to super hub-bottlenecks in our network. The hubs are in condensing interactions (Figure 2). All of 15 hubs are connected and the significant role of *INS* and *LEP* is highlighted (Figure 2-A). The action map of hubs is 88% similar to the map of hubs and bottlenecks (Figure 2-B). Bottlenecks did not show substantial contribution in the network. In other words, the hub genes showed to be more important than bottlenecks in our network strength as they play major part in associating with each other.

What is more, by either addition or deletion of the central nodes in the nested pathway networks, the super hub-bottlenecks would not lose their great power. On the other hand, the action type between super hub-bottlenecks shows that leptin, insulin, and *POMC* are widely in tight intercommunications and may act as a core in Anorexia Network. This fact is also in agreement with literature. Leptin and insulin are known as anorexigenic hormones and their regulatory effect on *POMC* showed to have fundamental role in appetite and energy balance in hypothalamus (23). Since the important role of obtained super-hub bottlenecks are well-established in AN, other hub-bottlenecks were also literature reviewed. In this sense, *GCG*, *SST*, and *ALB* as the lower ranked hub-bottlenecks showed some associations with AN (24-26). The findings from Figure 2 suggests deeper examination of isolated bottlenecks, tabulated in Table 2. The isolated genes are with very low rank of degree. This supports the fact that high degree (17) genes are more essential than bottlenecks in our network. Plasma leptin and insulin levels were decreased in the women with AN (27). Prominent role of insulin and also glucose in AN is a well-known fact emphasized by scientist in the about 30 yr ago (28).

Furthermore, regulation of behavior, regulation of carbohydrate biosynthetic process, and regulation of appetite are the foremost predicted BPs for our hub genes (Figures 3 and 4). Any alteration in the hub genes may disrupt the underlying processes in Figure 3. Similarly, the principal MFs for these hubs are extracted as neuropeptide receptor binding, neuropeptide hormone activity, and hormone activity. Thus, the prominent contribution of hormones and also behavioral alteration in AN is highlighted in this study. It seems beside feeding and metabolic management, behavioral treatment is necessary to reduce problems of patients. This finding consists of the report which introduces cognitive behavioral therapy and interpersonal psychotherapy as effective treatment methods for anorexia (29). 

Based on PPI network analysis, our suggested panel including *LEP, INS, POMC, GCG, SST*, and *ALB* are more prominent than the other contributing genes in AN. This panel and relevant BPs and MF reflect the required therapeutic methods and management of AN disorder. 

**In conclusion, **the nominated central genes in our network may have a notable contribution in anorexia pathogenicity and management. The finding pointed to the hormonal and behavioral features of AN. Nevertheless, the validation methods especially, experimental assessments should be applied to support this claim. 
